# Clinical characteristics and viral etiologies of outpatients with acute respiratory infections in Huzhou of China: a retrospective study

**DOI:** 10.1186/s12879-018-3668-6

**Published:** 2019-01-08

**Authors:** Xiaohong Wen, Qiuling Huang, Hong Tao, Weihua Zou, Min Gao, Huihui Guo, Xing Yao, Dawei Cui, Xiang Wang

**Affiliations:** 1 The First People’s Hospital Affiliated to Huzhou University, Huzhou, 313000 China; 2 Department of Clinical Laboratory, Huzhou Central Hospital, Huzhou, 313000 China; 3 Department of Laboratory & Pharmacy, Suzhou Vocational Health College, Suzhou, 215009 China; 4 Department of Blood Transfusion, the First Affiliated Hospital, College of Medicine, Zhejiang University, Hangzhou, 310003 China

**Keywords:** Acute respiratory infections, Viral etiologies, Coinfections, Clinical characteristics

## Abstract

**Background:**

Viruses are commonly found in patients with acute respiratory infections (ARIs). However, the viral etiologies and clinical characteristics of outpatients with ARIs are poorly understood in China. Here, we identified the viral etiologies in outpatients with ARIs in Huzhou, China.

**Results:**

Our results indicated that of 426 outpatients, 246 were positive for viruses. Of them, 221 were positive for a single virus, including influenza A, which comprised H3N2 (28.5%) and pandemic H1N1 (2009) (19.0%), enterovirus (10.4%), and influenza B (8.6%). Other single viruses were detected at less than 8.0%. Twenty-five patients were positively coinfected with two viruses. The prevalent viruses in coinfections were rhinovirus and H3N2 virus (28.0%). Viruses were major pathogens in young children (< 5 years) (75.0%). Coinfections were prevalent in older adults (11.9%) and young children (9.5%). Virus-positive outpatients presented higher temperatures and more sore throat, fatigue and shortness of breath than virus-negative outpatients. ARIs and most virus detections peaked during the winter, but enteroviruses emerged between April and September.

**Conclusion:**

Viruses are major agents of ARIs among outpatients in Huzhou, China. There was a variation in the distribution of viruses across different age groups and seasons. These findings are beneficial for planning prevention and treatment services for outpatients with ARIs.

## Background

Acute respiratory infections (ARIs) are common and major public health threats, causing high morbidity and mortality worldwide, particularly in developing countries [1, 2]. Many pathogens can result in ARIs, and viruses have been identified as major causes in ARIs among various populations; the most common viruses of ARIs include influenza A and B virus (FluA and FluB), rhinovirus (RhV), respiratory syncytial virus (RSV), parainfluenza virus (PIV) type 1–4, enterovirus (EV), adenovirus (ADV), human metapneumovirus (hMPV), human bocavirus (BoV), and coronavirus (CoV)-229E, NL63, OC43 and HKU1 [3–8].

Currently, there are few available vaccines to prevent respiratory virus infections [5, 6]. It is important to investigate the epidemic viral etiologies of ARIs to efficiently prevent and control viral epidemics in the future. It is well known that the early and rapid molecular detection of respiratory viruses is valuable to prevent and control ARIs [7, 8]. However, only a portion of patients with ARIs have their viral etiologies detected because of the expensive testing costs in China and other developing countries [3, 5, 9, 10]. Moreover, the spectrum of viral etiologies is closely correlated with various factors, such as age, season, geographical region, medical condition, and immune status [4, 6, 10–13].

In this study, we collected clinical and demographic data from outpatients including children and adults with ARIs, and their specimens were tested for 15 viruses. This study aims to provide basic data to direct local disease prevention and control measures for ARIs in Huzhou, China.

## Methods

### Study design

This study was conducted from January 2015 to April 2016 in two general hospitals in Huzhou city located beside Tai Lake of southeast China. Demographic and clinical data from all enrolled ARIs outpatients of any age were collected, and clinical specimens from the upper respiratory tract of these outpatients were tested for 15 viruses by the multiplex RT-PCR method using a Seeplex® RV15 ACE Detection Kit (Seegene, Korea). Furthermore, a positive sample with FluA virus was discriminated for seasonal H1N1 (sH1N1), seasonal H3N2 (sH3N2) and pandemic H1N1 (2009) viruses by a one-step real-time RT-PCR assay from Shanghai ZJ Bio-Tech Co., Ltd. (Shanghai, China).

### Patients and clinical samples

Outpatients of any age were enrolled from January 2015 to April 2016 at the Department of Fever Outpatient Clinic of the First People’s Hospital of Huzhou and at the Huzhou Central Hospital, Huzhou, China. Outpatients suffering from fever now or in recent days, such as influenza-like illness (ILI) and ARIs patients, were seen at the fever outpatient clinic. A case definition of ARI was described in a previous report [9]. Briefly, patients with ARIs presenting with at least one of the following symptoms: cough, sore throat, shortness of breath or coryza as an acute onset of symptoms within 7 days were judged by a clinician for an infection. Clinical specimens from the upper respiratory tract of patients, including throat swabs, nasal aspirates and washes, or sputum specimens, were collected and kept in 3 mL of viral transport medium stored at − 80 °C until testing for respiratory viruses. Clinical data including age, oral body temperature, clinical symptoms and other information were recorded in case report forms during face-to-face interviews.

### Laboratory analysis

Viral nucleic acids were extracted from 200 μl of clinical samples using the RNeasy Mini Kit (Qiagen, Valencia, CA) and eluted in 50 μl of elution buffer according to the manufacturer’s protocol. cDNA synthesis was conducted with a PrimeScript™ II 1st Strand cDNA Synthesis Kit (Takara, Dalian, China). The specimens were detected for viruses by the multiplex RT-PCR method using a Seeplex® RV15 ACE Detection Kit (Seegene, Korea) according to the manufacturer’s instructions. The following 15 viruses were tested in three groups: Group A: ADV, PIV-1, PIV-2, PIV-3, and CoV-229E/NL63; Group B: CoV-OC43/HKU1, RhV, RSV-A, RSV-B, and FluA; Group C: BoV, FluB, hMPV, PIV-4 and EV. The full meaning of each of the viruses abbreviated thus was defined as follows: ADV, PIV-1, PIV-2, PIV-3, CoV-229E/NL63, CoV-OC43/HKU1, RhV), RSV-A, RSV-B, FluA, human BoV, FluB, hMPV, PIV-4, and EV. Additionally, samples positive for FluA virus were further discriminated for sH1N1, sH3N2 and pandemic H1N1 (2009) viruses by a one-step real-time RT-PCR assay from Shanghai ZJ Bio-Tech Co., Ltd. (Shanghai, China).

### Statistical analysis

Statistical analysis was conducted with SPSS software (v18.0, SPSS, Chicago, IL, USA). Descriptive statistics were used to analyze the seasonal and age distribution and infection rates of different respiratory viruses. Chi-squared tests were used to compare different age groups, gender, and clinical characteristics between age virus-positive and virus-negative outpatients with ARIs. *P*-values < 0.05 were considered to be statistically significant.

## Results

### Viral etiologies of ARIs outpatients

**Fig. 1:**
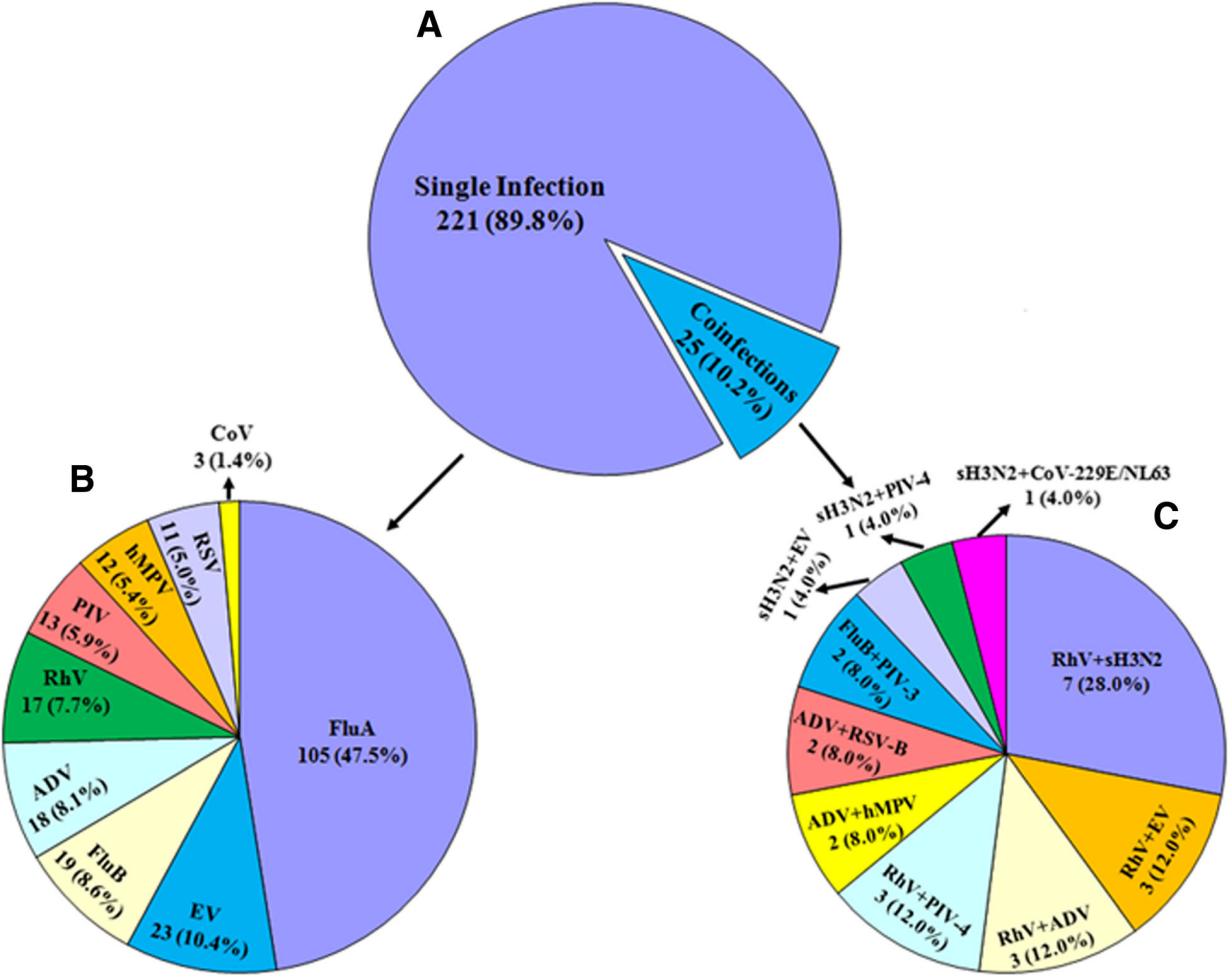
Identification of viral etiologies based on 246 virus-positive outpatients with ARIs. **a** Single viral infection and viral coinfections. **b** Distribution of the 221 cases with single viral infection. **c** Distribution of different combinations of the 25 cases with viral coinfections

A total of 426 outpatients with ARIs were enrolled from January 2015 to April 2016 in this study. Of them, 246 specimens (57.7%, 246/426) were positive for at least one virus, and single infections accounted for 89.8% (221/246) of cases. Coinfections were observed in 10.2% (25/246) of cases (Fig. 1a). Of the single virus infections, FluA virus was the most frequent virus, identified in 47.5% (105/221) of the cases, comprising 63 (60.0%) cases of sH3N2 virus and 42 (40.0%) cases of pandemic H1N1 (2009) virus patients, followed by EV (10.4%, 23/221), FluB (8.6%, 19/221), ADV (8.1%, 18/221), RhV (7.7%, 17/221), hMPV (5.4%, 12/221), and other viruses that were identified under 5.0%, respectively (Fig. 1b). Of the 25 coinfections, RhV + sH3N2 viruses were predominantly identified and accounted for 28.0% (7/25) of cases. RhV + EV, RhV + ADV and RhV + PIV-4 viruses equally accounted for 12.0% (3/25) of cases. ADV + hMPV, ADV + RSV-B, and FluB+PIV-3 also equally accounted for 8.0% (2/25) of cases. Other coinfections were identified in 4.0% (1/25) of cases (Fig. 1c). sH1N1 virus was not detected in this study.

### Clinical characteristics of outpatients with ARIs

Demographic and clinical characteristics of outpatients with ARIs are shown in Table 1. Of 426 outpatients with ARIs, 234 (54.9%) were males, and 192 (45.1%) were females. The distribution of viruses did not significantly differ between males and females (χ^2^ = 1.836, *P* = 0.175). However, the distribution of viruses notably differed among the different age groups (χ^2^ = 28.668, *P* < 0.001). In addition, 90.1% (384/426) of ARIs outpatients suffered from high fever (body temperature ≥ 38 °C), followed by cough (68.5%), sore throat (51.9%), fatigue (45.8%), and other respiratory symptoms. Moreover, a significant difference was observed in clinical symptoms including fever (χ^2^ = 4.233, *P* = 0.0396), sore throat (χ^2^ = 26.82, *P* < 0.0001), fatigue (χ^2^ = 14.58, *P* < 0.0001), and shortness of breath (χ^2^ = 8.859, *P* = 0.0029) between virus-positive and virus-negative cases, and the proportion of patients with abdominal pain was greater among virus-negative cases (χ^2^ = 6.703, *P* = 0.0096). Additionally, physical examinations showed abnormal lung auscultation (12.9%) and X-rays (6.1%).

**Table 1:** Clinical characteristics of outpatients with ARIs by the presence of respiratory viruses

Characteristics	Total	Virus-positive	Virus-negative		*P* value
	*N* = 426	*N* = 246	*N* = 180	χ2	
	*n* (%)	*n* (%)	*n* (%)		
Gender					
Male	234 (54.9)	142 (60.7)	92 (39.3)	1.836	0.175
Female	192 (45.1)	104 (54.2)	88 (45.8)		
Age groups (years)					
< 5	84 (19.7)	63 (75.0)	21 (25.0)	28.668	0.000
5~18	139 (32.6)	89 (64.0)	50 (36.0)		
18~60	161 (37.8)	68 (42.2)	93 (57.8)		
≥60	42 (9.9)	26 (61.9)	16 (38.1)		
Clinical symptoms and physical examination					
Fever ≥38.0 °C	384 (90.1)	228 (59.4)	156 (40.6)	28.773	0.002
Cough	292 (68.5)	173 (59.2)	119 (40.8)		
Sore throat	221 (51.9)	154 (69.7)	67 (30.3)		
Fatigue	195 (45.8)	132 (67.7)	63 (32.3)		
Runny nose	137 (32.2)	85 (62.0)	52 (38.0)		
Sputum production	129 (30.3)	74 (57.4)	55 (42.6)		
Headache	87 (20.4)	52 (59.8)	35 (40.2)		
Shortness of breath	125 (29.3)	86 (68.8)	39 (31.2)		
Diarrhea	35 (8.2)	15 (42.9)	20 (57.1)		
Abdominal pain	33 (7.7)	12 (36.4)	21 (63.6)		
Lung auscultation	55 (12.9)	34 (61.8)	21 (38.2)		
Abnormal X-ray	26 (6.1)	17 (65.4)	9 (34.6)		

ARIs, acute respiratory infections

### Age distribution of different viruses

The distribution of the viral etiologies of the four age groups is shown in Table 2. The highest proportion was observed in young children (75.0%), and lowest proportion was observed in adults 18–60 years of age (42.2%). Similarly, the positive rate of cases with a single virus infection was highest in the young children (65.5%) and lowest in adults of 18–60 years of age (38.5%). Moreover, the positive rate of the cases with coinfections was highest in older adults (11.9%), followed by young children (9.5%). The predominant viruses among four age groups differed. FluA virus and subtypes were the most prevalent viruses in the older adults (≥60 years), and the converse was true in the young children (< 5 years). EV, RSV and PIV viruses predominated in the young children (< 5 years), and FluB, RhV and ADV viruses were more prevalent in the young adults (5~18 years).

**Table 2:** Age distribution of viruses from outpatients with ARIs

Viruses	< 5 years (*N* = 84) (%)	5~18 years (*N* = 139) (%)	18~60 years (*N* = 161) (%)	≥60 years (*N* = 42) (%)	Total (*N* = 426) (%)
Single infection	55 (65.5)	83 (59.7)	62 (38.5)	21 (50.0)	221 (51.9)
FluA	13 (15.5)	36 (25.9)	40 (24.8)	16 (38.1)	105 (24.6)
sH1N1	0 (0)	0 (0)	0 (0)	0 (0)	0 (0)
sH3N2	10 (11.9)	21 (15.1)	25 (15.5)	7 (16.7)	63 (14.8)
H1N1(2009)	3 (3.6)	15 (10.8)	15 (9.3)	9 (21.4)	42 (9.9)
EV	15 (17.9)	5 (3.6)	2 (1.2)	1 (2.4)	23 (5.4)
FluB	4 (4.8)	11 (7.9)	4 (2.5)	0 (0)	19 (4.5)
ADV	2 (2.4)	9 (6.5)	7 (4.3)	0 (0)	18 (4.2)
RhV	3 (3.6)	9 (6.5)	3 (1.9)	2 (4.8)	17 (4.0)
PIV	8 (9.6)	3 (2.1)	2 (1.2)	0 (0)	13 (3.0)
PIV-1	4 (4.8)	0 (0)	0 (0)	0 (0)	4 (0.9)
PIV-2	3 (3.6)	2 (1.4)	2 (1.2)	0 (0)	7 (1.6)
PIV-3	1 (1.2)	1 (0.7)	0 (0)	0 (0)	2 (0.5)
PIV-4	0 (0)	0 (0)	0 (0)	0 (0)	0 (0)
hMPV	4 (4.8)	7 (5.0)	0 (0)	1 (2.4)	12 (2.8)
RSV	6 (7.2)	2 (1.4)	2 (1.2)	1 (2.4)	11 (2.5)
RSV-A	3 (3.6)	1 (0.7)	0 (0)	0 (0)	4 (0.9)
RSV-B	3 (3.6)	1 (0.7)	2 (1.2)	1 (2.4)	7 (1.6)
Cov	0 (0)	1 (0.7)	2 (1.2)	0 (0)	3 (0.7)
CoV-OC43/HKU1	0 (0)	1 (0.7)	2 (1.2)	0 (0)	3 (0.7)
CoV-229E/NL63	0 (0)	0 (0)	0 (0)	0 (0)	0 (0)
BoV	0 (0)	0 (0)	0 (0)	0 (0)	0 (0)
Coinfections	8 (9.5)	6 (4.3)	6 (3.7)	5 (11.9)	25 (5.9)
RhV + sH3N2	3 (3.6)	1 (0.7)	1 (0.6)	2 (4.8)	7 (1.6)
RhV + EV	0 (0)	3 (2.2)	0 (0)	0 (0)	3 (0.7)
RhV + ADV	0 (0)	0 (0)	3 (1.9)	0 (0)	3 (0.7)
RhV + PIV-4	0 (0)	1 (0.7)	2 (1.2)	0 (0)	3 (0.7)
ADV + hMPV	2 (2.4)	0 (0)	0 (0)	0 (0)	2 (0.5)
ADV + RSV-B	2 (2.4)	0 (0)	0 (0)	0 (0)	2 (0.5)
FluB+PIV-3	0 (0)	0 (0)	0 (0)	2 (4.8)	2 (0.5)
sH3N2 + CoV-229E/NL63	1 (1.2)	0 (0)	0 (0)	0 (0)	1 (0.2)
sH3N2 + PIV-4	0 (0)	1 (0.7)	0 (0)	0 (0)	1 (0.2)
sH3N2 + EV	0 (0)	0 (0)	0 (0)	1 (2.4)	1 (0.2)
Total positive cases	63 (75.0)	89 (64.0)	68 (42.2)	26 (61.9)	246 (57.7)

ARIs, acute respiratory infections

### Seasonal distribution of different viruses

**Fig. 2:**
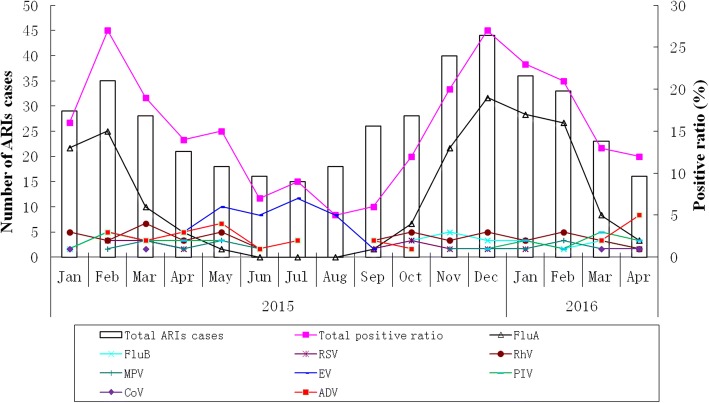
Seasonal distribution (months) of outpatients with ARIs caused by viruses FluA viruses consisted of seasonal H1N1 (sH1N1), seasonal H3N2 (sH3N2) and pandemic H1N1 (2009); RSV viruses included RSV-A and RSV-B subtypes; PIV viruses included PIV-1, PIV-2, PIV-3, PIV-4 subtypes; CoV viruses included CoV-OC43, CoV-HKU1, CoV-229E, and CoV-NL63 subtypes. BoV virus was not detected in this study.

The virus detection rates for different seasons are shown in Fig. 2. The proportion of positive viruses exhibited two waves corresponding to winter and spring, including Jan to Mar 2015, and Nov 2015 to Feb 2016. Similarly, the FluA virus also occurred more frequently in winter and spring. Conversely, EV infections were predominant between April and September. Other viruses occurred almost sporadically throughout the year without obvious seasonal trends, and a small number of ARI outpatients with virus infection were observed between June and September, excluding EV infections.

## Discussion

Viruses are major agents contributing to the high morbidity and mortality of patients with ARIs, particularly in children under 5 years of age [3, 4, 6]. Many reports describe the etiology and epidemiology of hospitalized ARIs patients, including children and/or adults worldwide [2, 14–16], although the study of outpatient ARIs in children and adults is more limited [3, 15]. In this study, some clinical characteristics significantly differed between virus-positive and virus-negative outpatient ARIs, such as fever, cough, sore throat, fatigue, and other respiratory symptoms. These findings implied that viruses were the most common causes of ARIs, easily eliciting severe clinical symptoms. Similar results were found in previous reports [10, 17–20].

Laboratory diagnosis of viruses is commonly conducted by conventional methods (such as culture or antigen detections), and real-time and multiplex RT-PCR assays have been considered to be important tools for identifying the etiologies of ARIs [3, 5, 7, 8]. Recently, a commercial multiplex PCR assay with a Seeplex® RV15 ACE Detection Kit was used to simultaneously and precisely identify the 15 viruses of ARIs in many laboratories [21–24]. The methods used in this study expanded and improved the capacity for testing viruses (15 viruses and 3 subtypes of FluA virus). In this study, 57.7% were positive for at least one virus, which was similar to the morbidity rates reported in previous studies in Pittsburgh (59.7%) and Vitória of Southeast Brazil (54.3%) [15, 25], but was different in China and compared with other reports [1, 3–5, 24, 26]. The single infection (89.8%) was predominant in our study, particularly the FluA virus (47.5%), which was consistent with previous reports in Shandong Province, Beijing, of China and other countries [5, 25–29]. sH3N2 and RhV coinfections were predominant among viral coinfections, which was different from previous reports [5, 15, 24, 29, 30]. The discrepancy of the predominant coinfections might be closely associated with principal epidemical viruses in the local region.

The proportion of respiratory viruses notably differed across different age groups; the virus positive rate was the highest in young children under 5 years but was lowest in adults (18~60 years) in this study. These findings indicated that the viruses were the predominant pathogen found in young children with ARIs, and similar morbidity rates have been reported in previous studies [5, 6], although the morbidity rate in this study also differed from those in other studies [28]. The Flu A virus was the predominant virus among the three age groups, excluding young children, and was highest among older adults (≥60 years) and lowest among young children; however, EV was the highest in young children among all age groups. Some studies show that EVs and RhVs can be difficult to discriminate with RT-PCR primers unless accompanied by amplicon sequencing, and FluA virus and RhV might fail to detect RhV due to competition between the amplification reactions [21–23]. Therefore, all positive RhV and/or EV specimens and 10 FluA virus specimens with random selection were identified by sequencing assay, respectively, and among them, four RhV positive and 3 EV positive specimens were not sequenced due to low viral load in the specimens. Additionally, viral coinfections predominantly occurred in young children (< 5 years) and older adults (≥60 years), which was consistent with previous reports in China and other countries [1, 5, 6, 15, 26, 28]. These results indicated that the detection rate of viruses in ARIs was closely associated with the age of outpatients because age affects immune status, exposure opportunities to viruses, and other lifestyle behaviors of people. Generally, young children and older adults have weak immune systems against viruses, which might contribute to their higher susceptibility to viruses than young adults and adults who have strong immune status against viruses [12, 13]. Moreover, young children have more opportunities for exposure to EVs than young adults and adults, which might cause higher incidences of EV infection than in other age groups [3, 24, 28].

Many studies have indicated that viral ARIs are affected by seasonal distributions and occur commonly in spring, autumn, and winter [3, 5, 15, 20, 27, 29]. In this study, viruses from outpatient ARIs were detected throughout the whole year and commonly occurred in spring and winter, with peaks occurring in February and December 2015. Similar results were reported in other studies [20, 27, 29]. Moreover, our results demonstrated that EV infections occurred between April and September 2015, with the peak detection rate in August 2015, which was in accordance with previous reports [27]. Many studies have shown that EVs are often detected in summer and autumn [27, 31–34]. We speculate that climate conditions might be an important factor for EV detection in temperate regions. These findings indicated that the geographical diversity of surveillance areas with warm and wet climate conditions beside Tai Lake might contribute to the variability in seasonal trends and viral etiologies of ARIs.

Our study had some limitations. First, there were only 426 outpatients with ARIs enrolled in the two local hospitals; because this was a small sample size, it may be difficult to estimate the disease severity of outpatient ARIs in detail. More large-scale surveillance for ARIs will performed, and analyses over additional years will provide a more accurate picture of seasonal variation in respiratory virus circulation in this community in the future. Second, we detected only viruses, but did not test for bacterial pathogens in the respiratory tracts of outpatients with ARIs; therefore, our data regarding pathogens causing ARIs were not comprehensive, which also affected our ability to determine the relationships between pathogens and disease severity in outpatients with ARIs. Taken together, our results were valuable to a certain degree for assessing outpatients and clinical treatments.

## Conclusions

In summary, this study provides important epidemiologic data regarding the clinical characteristics, viral spectrum, age distribution and seasonality of viruses in outpatients with ARIs in Huzhou, China. These findings contribute to evaluating the burden of virus infections in outpatients, including young children and adults. Timely and accurate diagnosis of pathogens in outpatients with ARIs is required to reduce the burden caused by these diseases.

## Abbreviations

ADV: Adenovirus
ARIs: Acute respiratory infections
BoV: Bocavirus
CoV: Coronavirus
EV: Enterovirus
Flu: Influenza
hMPV: Human metapneumovirus
RhV: Rhinovirus
RSV: Respiratory syncytial virus
